# Quality of life in patients aged 80 or over after ICU discharge

**DOI:** 10.1186/cc8231

**Published:** 2010-01-08

**Authors:** Alexis Tabah, Francois Philippart, Jean Francois Timsit, Vincent Willems, Adrien Français, Alain Leplège, Jean Carlet, Cédric Bruel, Benoit Misset, Maité Garrouste-Orgeas

**Affiliations:** 1 Medical-Surgical ICU, Saint Joseph Hospital Network, 185 rue Raymond Losserand, 75014 Paris, France; 2 Cytokines and inflammation unit, Institut Pasteur, 28 rue du Docteur Roux, 75015 Paris, France; 3 INSERM U823 "Epidemiology of cancers and severe diseases", Albert Bonniot Institute, Rond-point de la Chantourne, 38706 La Tronche Cedex; 4 Medical Intensive Care Unit, Albert Michallon Teaching Hospital, Joseph Fournier University, BP 217, 38043 Grenoble cedex 09, France; 5 Recherche épistémologiques et historiques sur les sciences exactes et les institutions scientifiques (REHSEIS), UMR 7596, Université Paris Diderot, Paris VII, 5 rue Thomas Mann, 75205 Paris Cedex 13, France; 6 University Paris Descartes, 12 rue de l'école de médecine, 75005 Paris, France

## Abstract

**Introduction:**

Our objective was to describe self-sufficiency and quality of life one year after intensive care unit (ICU) discharge of patients aged 80 years or over.

**Methods:**

We performed a prospective observational study in a medical-surgical ICU in a tertiary non-university hospital. We included patients aged 80 or over at ICU admission in 2005 or 2006 and we recorded age, admission diagnosis, intensity of care, and severity of acute and chronic illnesses, as well as ICU, hospital, and one-year mortality rates. Self-sufficiency (Katz Index of Activities of Daily Living) was assessed at ICU admission and one year after ICU discharge. Quality of life (WHO-QOL OLD and WHO-QOL BREF) was assessed one year after ICU discharge.

**Results:**

Of the 115 consecutive patients aged 80 or over (18.2% of admitted patients), 106 were included. Mean age was 84 ± 3 years (range, 80 to 92). Mortality was 40/106 (37%) at ICU discharge, 48/106 (45.2%) at hospital discharge, and 73/106 (68.9%) one year after ICU discharge. In the 23 patients evaluated after one year, self-sufficiency was unchanged compared to the pre-admission status. Quality of life evaluations after one year showed that physical health, sensory abilities, self-sufficiency, and social participation had slightly worse ratings than the other domains, whereas social relationships, environment, and fear of death and dying had the best ratings. Compared to an age- and sex-matched sample of the general population, our cohort had better ratings for psychological health, social relationships, and environment, less fear of death and dying, better expectations about past, present, and future activities and better intimacy (friendship and love).

**Conclusions:**

Among patients aged 80 or over who were selected at ICU admission, 80% were self-sufficient for activities of daily living one year after ICU discharge, 31% were alive, with no change in self-sufficiency and with similar quality of life to that of the general population matched on age and sex. However, these results must be interpreted cautiously due to the small sample of survivors.

## Introduction

The human lifespan is increasing across the world as a result of economic progress, technological advances, and improved healthcare. In 2007, it was estimated that 98 million people, or 1.5% of the world population, were older than 80 years [1]. French census data show a steady increase in the proportion of elderly individuals and, in 2008, 3.9 million individuals were aged 75 to 84 years and 1.4 million were older than 85 years [2].

One consequence of this increasing lifespan is that a growing number of very elderly patients are being admitted to the intensive care unit (ICU). Critical care seeks not only to ensure survival, but also to restore the pre-admission level of function and to return the patient to his or her pre-admission living arrangements. Elderly patients who survive a critical illness at the cost of further functional impairments may require nursing-home admission, an outcome most of them deem undesirable [3]. Whereas self-sufficiency is an objective outcome, quality-of-life assessments provide information on outcomes perceived by ICU survivors [4]. The World Health Organization (WHO) defines quality of life as 'an individual's perception of their position in life in the context of the culture and value systems in which they live and in relation to their goals, expectations, standards and concerns' [5].

Few data are available on quality of life in very elderly ICU survivors compared to the general population [6-8]. One study detected no difference [8], another found decreases in specific domains with similar overall quality of life [6], and two studies found worse quality of life [7,9]. These discrepancies may be ascribable to differences in the tools used to assess quality of life and to the use of tools designed for the general population that may be inappropriate in the very old [10].

The aim of this study was to evaluate self-sufficiency and quality of life one year after ICU discharge in patients aged 80 years or over. Quality of life was assessed using a validated tool developed for the elderly by the World Health Organization.

## Materials and methods

### Setting

The study was performed at the Saint Joseph Hospital, a 460-bed tertiary-care non-university hospital for adults, located in Paris, France. The hospital provides services in all the medical specialties and in all fields of surgery except neurosurgery. The ICU is a 10-bed medical unit that admits about 400 patients per year (mean age, 62 years), of whom 70% have medical conditions. In our ICU, we have no predefined admission criteria. Our triage process has been described elsewhere [9].

### Patients

From January 1, 2005, to December 31, 2006, we included all patients aged 80 years or over at ICU admission. Patients who were admitted several times during the study period had only their first stay included in the study. For each patient, the attending intensivist completed a case-report form in a database using data-capture software (RHEA, Outcomerea, Rosny Sous Bois, France). The following information was recorded prospectively: age and sex; admission category (medical, scheduled surgery, or unscheduled surgery); invasive procedures (number of arterial and/or venous central lines, endotracheal and noninvasive ventilation, dialysis, and tracheotomy); use of vasoactive agents and inotropic support; and patient location prior to ICU admission (with *transfer from wards *defined as being in the same hospital or another hospital before ICU admission). Nine reasons for ICU admission were defined prospectively before the study (respiratory failure, heart failure, renal failure, coma, multiple organ failure, chronic obstructive pulmonary disease, monitoring, trauma, and scheduled surgery). Co-morbidities were assessed using the McCabe score [11] and the Knaus classification system [12]. The McCabe score distinguishes two categories of underlying diseases based on whether death is likely to occur within five years or within one year [11]. Severity of the acute illness and organ dysfunction were measured at ICU admission using the Simplified Acute Physiology Score (SAPS II) [13], the Logistic Organ Dysfunction (LOD) score [14], and the Sepsis-Related Organ Assessment (SOFA) [15]. Withholding and withdrawal decisions, which were made according to the recommendations of the Francophone Society for Critical Care (SRLF) [16], were recorded; as well as lengths of the stays in the ICU and acute-care hospital and vital status at ICU and hospital discharge.

### Quality of life

Information on prior self-sufficiency was obtained from the patient or family members, either at admission or within the first few days after admission, according to standard practice in our unit. Self-sufficiency was evaluated using the modified Katz Index of Activities of Daily Living (ADL), which assesses the ability to perform six basic daily activities (bathing, dressing, toileting, transferring, continence, and feeding) on a seven-point scale where zero indicates complete dependence and six complete independence [17].

Long-term quality of life was assessed using the WHOQOL-BREF and WHOQOL-OLD questionnaires developed by the World Health Organization (WHO) [18,19]. The WHOQOL-BREF, which is the abbreviated version of the WHOQOL-100 [19], is a cross-culturally developed and validated questionnaire that can be used in specific cultural settings to collect data suitable for subsequent comparison across cultures. It has 26 items that cover four domains: physical health, psychological health, social relationships, and environment. It also measures the individual's perceptions of quality of life and health via two items ('How would you rate your quality of life?' and 'How satisfied are you with your health?'), each rated from 1 (very poor/dissatisfied) to 5 (very good/satisfied). The WHOQOL-OLD was developed as an add-on module that can be used with other WHOQOL instruments to specifically address important facets of quality of life in older adults [18]. It has 24 items that cover six facets (sensory abilities; autonomy; past, present, and future activities; social participation; death and dying; and intimacy). The WHOQOL-BREF questionnaire is available on the web [20] and from national WHO field centers. Domain scores are calculated from the items then converted to an overall percentile scale that ranges from very poor (0%) to very good (100%).

### Follow-up measures

Outcomes one year after ICU discharge were assessed over the phone. Patients who failed to answer the first call were called again on different days, for a total of four calls. When we were unable to contact the patient by phone, we sought vital status information by calling the primary care physician and by looking for a death certificate at the appropriate registry office (or consulate if the patient was not French). The Katz Index and the WHOQOL-OLD and WHOQOL-BREF questionnaires were completed during a telephone interview conducted by one of us (AT). Because quality of life is a subjective personal concept that cannot be readily evaluated by relatives, only the patients completed the quality-of-life questionnaires. In contrast, relatives were asked for information on self-sufficiency that could not be obtained from the patients. The institutional review board waived requirement for written informed consent at ICU admission. Each patient received information about their inclusion in the study at ICU discharge and at the beginning of the phone call, and then asked to consent to the interview.

### Statistical analysis

Quantitative data are reported as mean ± SD if normally distributed and as median (interquartile range (IQR)) otherwise. Qualitative data are reported as n (%). WHOQOL scores were calculated using the files created in SPSS by the WHO. The control group was a random sample of the general population matched on age and sex to our patients and derived from the sample used to validate the French version of the WHOQOL-OLD. Comparisons of self-sufficiency before and after the ICU stay and comparisons of WHOQOL scores after the ICU stay in our patients and in the general population were done using the Wilcoxon test for paired data. Statistical analyses were performed using SAS software (SAS 9.1, SAS Institute, Cary, NC, USA).

## Results

### Patients

During the two-year study period, among the 630 consecutive admissions to our ICU, 115 (18.2%) were for patients aged 80 years or over (mean age, 84 ± 3 years; range, 80 to 92). There were seven readmissions (one patient readmitted twice and five patients readmitted once each, of whom two were alive after one year and completed our evaluation). We excluded two patients with missing data, which left 106 patients for the study. These patients had a mean age of 84 ± 2 years. Among them, 69 (65.1%) had medical conditions, 21 (19.8%) required unscheduled surgery, and 68 (64%) were transferred from wards. At admission, the mean Simplified Acute Physiology Score was 45 ± 18.3 points and the mean Logistic Organ Dysfunction score was 5.4 ± 3.5 points. During the ICU stay, 63 (59.4%) required ventilatory assistance, 48 (45.3%) epinephrine/norepinephrine, and 20 (18.9%) dialysis. The median ICU stay was six days (IQR, 3 to 11) and the median post-ICU hospital stay was eight days (IQR, 0 to 18.5).

Of the 106 patients, 40 (37.7%) died in the ICU and 39 (36.8%) had treatment-limitation decisions, which consisted in withholding life-support in 22 (20.8%) patients and withdrawing life support in 20 (18.9%) patients, with three patients having both categories of decisions.

### Follow-up and quality of life

Of the 66 (62.2%) patients discharged alive from the ICU, eight died before hospital discharge. Hospital mortality was 48/106 (45.2%). In addition, 25 patients died before the one-year evaluation. Thus, one-year mortality was 73/106 (68.9%). Of the 33 survivors at one year, seven refused the evaluation (two were unhappy with our institution, one stated having insufficient time, two had hearing loss, and two lived at home but did not answer our multiple calls). Of the 26 remaining patients, three had dementia that precluded them from completing the evaluation. Self-sufficiency in these three patients was assessed by the relatives; they had ADL scores of 4, 4, and 2, respectively. Quality of life was not assessed in these three patients.

Quality of life was therefore assessed in 23 patients, whose mean age was 84 ± 3 years; there were 17 (73.9%) males (Table 1). Mean time from ICU discharge to evaluation was 471 ± 121 days (25^th ^to 75^th^:375 to 583), due to difficulties experienced in locating some of the patients. Mean phone call duration was 42 ± 14 minutes. As shown in Table 2, self-sufficiency was not modified after the ICU stay compared to the pre-ICU status (median index values, 6 vs. 6, respectively). Table 3 compared quality-of-life data in the 23 patients and in the general population matched on sex and age. The survivors had significantly higher scores for psychological health; social relationships; environment; fear of death and dying; expectations about past, present, and future activities; and intimacy (friendship and love). Of the 23 patients, 18 (78%) said they would agree to another ICU admission should the need occur in the future.

**Table 1:** Main characteristics of survivors and nonsurvivors

Variables	Non survivors N = 83	Survivors N = 23	*P *value
**Age in years, mean ± SD (range)**	84 ± 3 (80 to 93)	84 ± 3 (80 to 92)	0.99
**Males, n (%)**	41 (49.4)	17 (73.9)	0.03
**Body mass index, kg/m^2^**	25.6 ± 5	24.1 ± 4	0.19
**McCabe classification, n (%)**			0.042
**Underlying disease: none or nonfatal**	32 (38.6)	14 (60.9)	
**Underlying disease expected to cause death within five years**	36 (43.4)	9 (39.1)	
**Underlying disease expected to cause death within one year**	15 (18.1)	0	
**Underlying diseases according to Knaus, n (%)**			
**At least one co-morbid condition**	38 (45.8)	7 (30.4)	0.18
**Hepatic**	1 (1.2)	0	
**Cardiovascular**	27 (32.5)	4 (17.4)	0.15
**Pulmonary**	19 (22.9)	3 (13)	0.30
**Renal**	9 (10.8)	1 (4.3)	0.34
**Immunosuppression**	3 (3.6)	1 (4.3)	0.87
**Patient location before ICU admission, n (%)**			
**Transfer from ward**	54 (65.1)	14 (60.9)	0.71
**Pre-ICU hospital stay,** **median (IQR)**	2 (0 - 6)	1 (0 - 5)	0.57
**Emergency room/home, n (%)**	29 (34.9)	9 (39.1)	0.71
**Admission category, n (%)**			0.27
**Medicine**	55 (66.3)	14 (60.9)	
**Unscheduled surgery**	14 (16.9)	7 (30.4)	
**Scheduled surgery**	14 (16.9)	2 (8.7)	
**Main symptom at admission, n (%)**			
**Septic shock and multiple organ failure**	16 (19.3)	8 (34.8)	0.11
**Other shock**	11 (13.3)	3 (13)	0.97
**Acute respiratory failure**	19 (22.9)	4 (17.4)	0.57
**Acute COPD exacerbation**	9 (10.8)	1 (4.3)	0.34
**Acute renal failure**	11 (13.3)	1 (4.3)	0.23
**Coma**	6 (7.2)	3 (13)	0.37
**Monitoring**	11 (13.3)	3 (13)	0.97
**Severity of illness at admission, Mean ± SD**			
**SAPS II**	47 ± 19.2	38.1 ± 12.7	0.047
**LOD**	5.7 ± 3.7	4.3 ± 2.5	0.10
**SOFA**	6.6 ± 3	5.7 ± 3	0.11
**Intensity of care, n (%)**			
**Endotracheal mechanical ventilation**	49 (59)	14 (60.9)	0.87
**Epinephrine/Norepinephrine**	38 (45.8)	10 (43.5)	0.84
**Dobutamine**	22 (26.5)	3 (13)	0.17
**Dialysis**	19 (22.9)	1 (4.3)	0.04
**Central venous catheter**	54 (65.1)	12 (52.2)	0.25
**Arterial catheter**	33 (39.8)	8 (34.8)	0.66
**Length of ICU stay, days, median (IQR)**	6 (3 - 12)	5 (3 - 9)	0.42

**Length of post-ICU hospital stay, median (IQR)**	1 (0 - 15)	17 (9 - 28)	0.0007

IQR = interquartile range; COPD = chronic obstructive pulmonary disease; SAPS II = Simplified Acute Physiologic Score [13]; LOD Logistic Organ Failure [14]; SOFA = Sepsis-Related Organ Assessment [15]

**Table 2:** Self-sufficiency before and after the ICU stay shown by percent of patients

	Nonsurvivors N = 83	Patients alive with one-year QOL data N = 23	
Self-sufficiency^1^		Before ICU admission	After one year^2^
**ADL = 6**	55 (66.3)	19 (82.6)	17 (74)
**ADL = 5**	4 (4.8)	1 (4.3)	2 (8.7)
**ADL = 4**	8 (9.6)	3 (13)	2 (8.7)
**ADL = 3**	1 (1.2)	0	1 (4.3)
**ADL = 2**	5 (6)	0	0
**ADL = 1**	2 (2.4)	0	1 (4.3)
**ADL = 0**	8 (9.6)	0	0
**Median ADL Score** **(IQR)**	6 (4 to 6)	6 (6 to 6)	6 (5 to 6)

^1^Self-sufficiency was assessed using the Katz Index of Activities of Daily Living (ADL) [17], with each activity being scored from zero (complete dependence) to six (complete independence).

QOL = quality of life; ICU = intensive care unit

^2^*P *= 0.80 for the comparison of self sufficiency one year after ICU discharge and before ICU admission in the 23 alive patients, for the whole activities of daily living

**Table 3:** Quality of life of the survivors compared to the general population

	Study population N = 23	General population matched on age and gender	P value
**QOL-BREF**			
**Overall perception of QOL**	73.9 ± 18.5	73 ± 19.6	0.87
**Overall perception of health**	72.7 ± 18.0	63.5 ± 21.4	0.12
**Physical health**	62.1 ± 16.6	56.7 ± 18	0.29
**Psychological health**	69.4 ± 16.3	56.5 ± 18.7	0.02
**Social relationships**	73.2 ± 16.7	60.2 ± 17.0	0.01
**Environment**	77.3 ± 12.1	67.5 ± 13.4	0.01
**QOL-OLD**			
**Sensory abilities**	64.7 ± 30.0	64.4 ± 18.4	0.96
**Autonomy**	63.6 ± 12.6	54 ± 22.6	0.08
**Death and dying**	77.9 ± 19.9	62.6 ± 23.1	0.02
**Past, present and future activities**	69.7 ± 17.7	57.6 ± 15.7	0.02
**Social participation**	60.5 ± 21.9	54.9 ± 18.1	0.35
**Intimacy**	68.2 ± 18.2	55.1 ± 20.3	0.03

QOL = quality of life; ICU = intensive care unit

QOL was assessed on a scale from 0 = very poor to 100 = very good

## Discussion

We found that patients aged 80 years or over who were selected for ICU admission had no change in self-sufficiency one year after ICU discharge compared to the pre-admission status and had similar quality of life compared to age-and sex-matched individuals from the general population. After one year, 78% of evaluated patients said they would agree to an ICU admission should they experience another critical illness.

During the study period, patients aged 80 years or over accounted for 18.2% of all patients admitted to our ICU. Patients in this age group were often refused ICU admission [9]. The 18.2% admission rate was in line with data in the French ICU Outcomerea database [21]. Mortality rates were high in our population: 37% at ICU discharge, 45.2% at hospital discharge, and 68.9% one year after ICU discharge. ICU and hospital mortality rates have varied across studies [9,22-26], probably because of case-mix differences. In contrast, one-year and two-year mortality rates have usually been within the 60% to 70% range [9,22-26], in line with our results. Our relatively high ICU mortality rate was explained by the large proportions of medical patients, patients transferred from other wards, patients with severe illness at admission requiring a high level of care not always provided to the very elderly [27], and treatment limitations during the ICU stay (40% of patients).

Self-sufficiency was not changed one year after ICU admission, in keeping with earlier data [6,8,9,24,25,28]. Furthermore, our patients had an overall good perception of their quality of life, comparable to that of the general population. On both quality-of-life questionnaires, mean scores on all facets were consistently within the 60% to 80% range. Physical health, sensory abilities, self-sufficiency, and social participation had slightly lower ratings than the other domains. Ratings were highest for social relationships, environment, and death and dying. Compared to an age-and sex-matched sample of the general population, our patients had better scores for psychological health; social relationships; environment; fear of death and dying; expectations about past, present, and future activities; and intimacy (friendship and love). One hypothesis is that surviving a life-threatening illness may offer opportunities for building psychological strength and diminishing the fear of death and dying. Moreover, patients probably adjust their expectations when faced with serious illness and disability, which may lead them to assign higher ratings to their quality of life. The results from this study must be interpreted cautiously due to the small sample and are at variance with those of our previous study in a similar population, in which quality of life was significantly poorer one year after ICU admission [9]. In this earlier study [9], quality of life was assessed using the modified Perceived Quality of Life scale and Nottingham Health Profile. Neither scale is specifically designed for older individuals. Therefore, the present study may provide a better assessment of quality of life. Both studies assessed self-sufficiency using the Katz Index of ADLs, and neither found any change after the ICU stay.

Most of the survivors said they would consent to ICU admission should they experience another acute life-threatening illness. The preferences of elderly patients regarding ICU admission are largely unknown in France and elsewhere, although surrogate designation is known to be popular in France [29]. Absence of a surrogate, or limited ability of the surrogate to predict the patient's wishes, may lead to ICU refusal of elderly patients who, if conscious, would choose ICU admission [30]. In our earlier study of patients aged 80 years or over, half the survivors said they would agree to another ICU admission [9], whereas the proportion was 72% in the present study. Differences in preferences of elderly patients may arise because of variations over time [31-33], most notably increased vulnerability [34] and family burden [35]. Patients who are in stable condition one year after an ICU stay may be more likely to express positive perceptions of their quality of life than patients with unstable disease. Furthermore, having experienced and survived an ICU stay may lead to a more positive opinion about ICU admission, compared to patients with no ICU experience. Patient preferences should be taken into account when deciding whether ICU admission is in order.

This study has several limitations. First, the data were obtained at a single center and may not be applicable to other ICUs. Second, the number of patients evaluated after one year was small. This limitation is ascribable to the usual high mortality rate in patients aged 80 years and over who require ICU admission. However, waiting one year to perform the assessment provides a sound estimate of post-ICU quality of life [4]. Third, our patients were selected for ICU admission based largely on self-sufficiency and on the expectation that life-supporting treatment would not prove futile. Our data may not apply to all patients aged 80 years and over who are admitted to the ICU, as admission policies vary widely across countries and within a given country. Furthermore, the patients evaluated in our study were long-term survivors and were willing to take the time to complete our evaluation.

## Conclusions

In a highly selected cohort of elderly patients, among whom fewer than one-third were alive one year after ICU discharge, self-sufficiency was unchanged one year after ICU admission and quality of life was comparable to that in the same-age general population. These results invite further investigations of the preferences of elderly patients regarding ICU admission. We are currently planning such a study.

## Key messages

• Patients aged 80 years or over who were admitted to the ICU were carefully selected based on self-sufficiency.

• Unlike previous studies, we found that one-year survival after ICU discharge was about 30%.

• In this small sample of survivors, one year after ICU discharge, the patients were satisfied with their level of self-sufficiency and quality of life.

• Quality of life, physical health, sensory abilities, self-sufficiency, and social participation had slightly lower ratings than other domains. Ratings were highest for social relationships, environment, and death and dying.

• Patient preferences should be taken into account when deciding whether ICU admission is in order.

## Abbreviations

ADL: activities of daily living; COPD: chronic obstructive pulmonary disease; ICU: Intensive care unit; IQR: interquartile range; LOD: logistic organ failure; SAPS II: Simplified Acute Physiologic Score II; SOFA: Sepsis-Related Organ Assessment; SPSS: Statistical Package for the Social Sciences; SRLF: Societé de Réanimation de Langue Française; WHO: World Health Organization; WHOQOL-100: World Health Organization-Quality of Life 100; WHOQOL-BREF: World Health Organization-Quality of Life BREF; WHOQOL-OLD: World Health Organization-Quality of Life OLD.

## Competing interests

The authors declare that they have no competing interests.

## Authors' contributions

AT collected the data and wrote the manuscript; MGO contributed to the design of the study and wrote the manuscript. JFT contributed to the design of the study, did the statistical analysis with responsibility for integrity of the data and the accuracy of the data analysis, and contributed to the final revision of the manuscript for important intellectual content. AF did the statistical analysis with responsibility for integrity of the data and the accuracy of the data analysis. AL contributed to the design of the study. FP, VW, JC, CB, and BM contributed to the final revision of the manuscript for important intellectual content. All the authors read and approved the final manuscript.
